# No Effects of a Brief Mindfulness Intervention on Controlled Motivation and Amotivation, but Effect Moderation Through Trait Mindfulness: a Randomized Controlled Trial

**DOI:** 10.1007/s12671-022-01968-7

**Published:** 2022-08-24

**Authors:** Sandra Oberleiter, Hannah Wainig, Martin Voracek, Ulrich S. Tran

**Affiliations:** Department of Cognition, Emotion, and Methods in Psychology, School of Psychology, University of Vienna, Liebiggasse 5, 1010 Vienna, Austria

**Keywords:** Motivation, Mindfulness, Self-determination theory, Effect moderation, RCT

## Abstract

**Objectives:**

Mindfulness is associated with the different forms of motivation according to self-determination theory (intrinsic, identified, and external motivation, and amotivation). However, causal evidence for reported negative associations of mindfulness with external motivation and amotivation is currently lacking. Therefore, this study investigated causal effects of a brief mindfulness intervention on motivation towards a personal goal. We differentiated distinct forms of motivation and also controlled for baseline motivation and trait mindfulness, which could act as a moderator of the interventional effects.

**Methods:**

Data of *N* = 91 participants were used, who were randomly assigned to either audio-guided meditation or a control condition. Situational motivation for a personal goal was assessed before and after the intervention. Trait mindfulness was measured with the Five Facet Mindfulness Questionnaire.

**Results:**

The intervention had a positive effect on the more autonomous forms of motivation (*d* = 0.48), which was, however, qualified by trait mindfulness; i.e., the effect was larger among participants low in trait mindfulness (*d* = 1.13 at 1 *SD* below the overall mean). There were no practically relevant effects on external motivation and amotivation.

**Conclusions:**

Mindfulness has a positive causal effect on more autonomous forms of motivation, but probably no relevant effects on external motivation and amotivation. Moderating effects of trait mindfulness need to be considered more systematically in this field of research, but also in research of mindfulness intervention more generally. Mindfulness interventions could be beneficially offered to persons low in trait mindfulness.

**Supplementary Information:**

The online version contains supplementary material available at 10.1007/s12671-022-01968-7.

The construct of mindfulness has received extensive public as well as scientific attention over the past two decades (Lee et al., 2021). It represents the awareness of the present moment without judging it (Brown & Ryan, 2003). Mindfulness induces attention to what is occurring both within the individual and in the environment and may thereby bring about greater autonomy and self-regulation. Yet, it may also lead to an increased awareness of internal conditions, such as feelings, impulses, and needs, and external stimuli, which can act as sources of pressure or temptation (Donald et al., 2020). Furthermore, some positive effects of mindfulness may also turn negative with increased practice. For example, there is a decrease of sleep duration and depth as well as an increase in cortisol arousal in correlation with a higher amount of practice time (> 30 min per day; Britton, 2019).

Mindfulness might also have further downsides rarely considered, such as a decrease in motivation. Rupprecht et al. (2019), for example, argue that mindfulness may affect motivation negatively because mindful individuals may be more aware of their self and their own personal values. This could make mindful individuals also more aware of toxic environments (e.g., in the work context) which do not align with their values, which, in turn, may lead to a decrease of motivation in such settings (Rupprecht et al., 2017; Walach et al., 2007). While this should actually be deemed a further beneficial effect of mindfulness, the focus of mindfulness and meditation on the present moment might arguably lower the motivation to perform tasks which have no immediate consequences. Hafenbrack and Vohs (2018) provided evidence for this idea with a series of five similarly designed experiments, wherein participants’ motivation for an instructed task was measured after performing a variety of brief interventions intended to induce a state of mindfulness. However, there are a number of issues which need to be addressed considering these results.

First, motivation is not a uniform construct. Self-determination theory (SDT; Ryan & Deci, 2000) differentiates various types of autonomous and controlled forms of motivation. “Autonomous” means acting with a feeling of volition and experiencing the feeling of choice. “Controlled” means acting under a certain pressure and the feeling of having to engage in the doing. Within these two umbrella terms, SDT differentiates five types of motivation: *Intrinsic motivation* is seen as the most autonomous form of motivation and represents engaging in tasks solely out of one’s own interest or enjoyment. *Identified motivation* is less autonomous and implies the willingness to participate in a task because it is seen as either valuable or rewarding. *External motivation* describes the most controlled form of motivation occurring when tasks are completed due to external punishments or rewards only. A weaker form of controlled motivation is the so-called *introjected motivation*, which describes behavior driven by incompletely internalized pressure or norms (however, introjected and external motivation are not further distinguished in the present study, as they are also not differentiated in widely used methods of assessment; see “Methods”). Lastly, *amotivation* is neither an autonomous nor controlled type of motivation, but rather depicts a missing intention to act. It often includes feeling ineffective and a lack of purpose (Donald et al., 2020).

SDT describes the construct of motivation as a continuum from controlled to autonomous, whereupon external, identified, and intrinsic motivation are characterized by their respective degree of internalization (Fig. 1). The process of internalization can be defined as the adoption of values, beliefs, and principles, whereby externally regulated behavior converts into internally regulated behavior. The stronger the internalization, the more autonomous is the motivated behavior (Gagné & Deci, 2005).

**Fig. 1:**
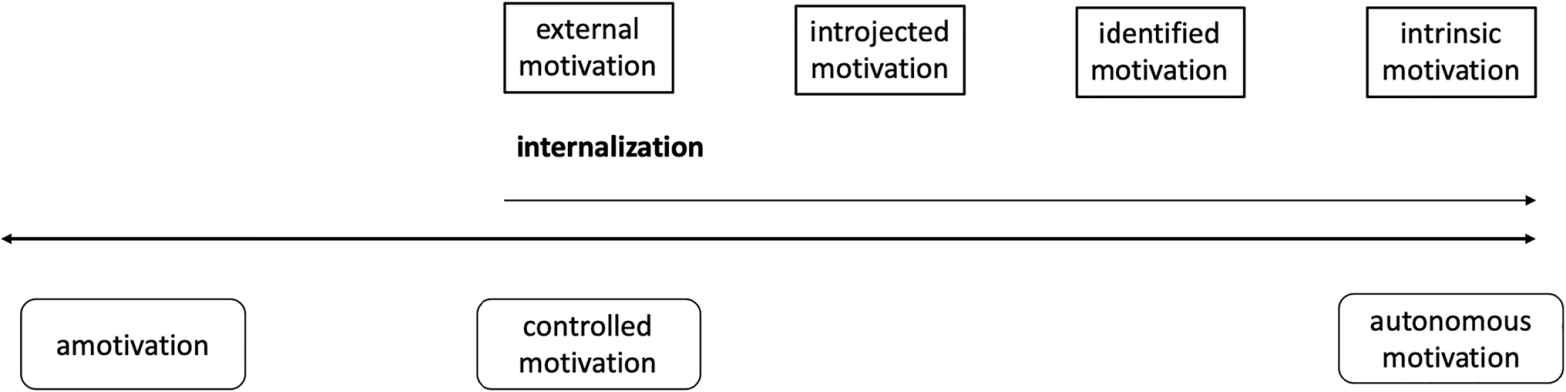
The motivation continuum according to self-determination-theory (Ryan & Deci, 2000), showing amotivation, controlled motivation, and the process of internalization to autonomous motivation. *Note*. Introjected motivation is featured in this figure, but was not differentiated empirically from identified motivation in the present study

Being mindful enables information to flow more freely and without judgment (Ryan et al., 2021). This makes potentially more information accessible that is needed for proper decision-making, which is why mindful processing may aid the action of internalization (but, presumably, may also lead to the detection of incongruous goals and, hence, to amotivation in such cases). Accordingly, Donald et al. (2020) predicted that mindfulness is positively associated with autonomous forms of motivation (intrinsic and identified), unrelated or negatively associated with controlled forms of motivation (external or introjected), and most negatively with amotivation. Mindfulness interventions were predicted to increase all forms of autonomous motivation and decrease all forms of controlled motivation. The meta-analysis of Donald et al. (2020), including 89 studies and involving more than 25,000 participants, indeed provided support for a positive association of mindfulness with autonomous forms of motivation (in both correlational and interventional studies) and a negative association with controlled forms of motivation and amotivation (based on correlational studies). However, intervention studies on controlled forms of motivation and amotivation are currently still lacking. Thus, it remains unclear whether the meta-analytically aggregated negative associations of mindfulness with controlled forms of motivation and amotivation might indeed be causal. The results of Hafenbrack and Vohs (2018) are not informative on this point, as their study did not differentiate the various forms of motivation.

Second, mindfulness can be conceptualized as both a state and a trait. The former is the individual experience of mindfulness in a certain situation or circumstance and is characterized by its transient nature. There is some evidence that even brief mindfulness interventions are sufficient to achieve a state of mindfulness that affects brain activity as well as cognition and feelings (Medvedev et al., 2017). On the other hand, recent research on MBSR (mindfulness-based stress reduction) has found that a minimum of 27 h of practice time is needed for a change in amygdala volume (Kral et al., 2022). These findings indicate larger neuronal changes for longer mindfulness interventions compared to only brief mindfulness interventions. Traits describe consistent characteristics or stable patterns of behavior of an individual. Yet, regular mindfulness practice has been shown to increase trait mindfulness over time which, in turn, may lead to a heightened sense of mindfulness in daily life, i.e., state mindfulness (Medvedev et al., 2017). Due to these ramifications, both state and trait mindfulness may need to be considered in studies of mindfulness interventions on motivation, besides differentiating for the various forms of motivation. Trait mindfulness likely moderates the effects of mindfulness interventions (larger effects for individuals low in trait mindfulness, smaller effects for individuals high in trait mindfulness). Hafenbrack and Vohs (2018), but also other extant studies, did not control for such moderating effects of trait mindfulness.

Third, individuals’ baseline motivation appears to be another important factor. Baseline motivation may affect the likelihood of study dropout (Coa & Patrick, 2016) and may also predict the individual benefit of an intervention (Peterson et al., 2006). Yet, in general, baseline measures are needed to separate the intervention effects from the effects of the comparison condition (Smyth & Milyavskaya, 2021) and to raise the internal and statistical conclusion validity of the study. Hafenbrack and Vohs (2018) did not assess and control for baseline motivation in evaluating the effects of mindfulness interventions on motivation, as is the case for many other studies in this field of research.

Fourth, a final important aspect to consider is the task or goal itself for which motivation is assessed. Mindfulness may especially motivate individuals to take on tasks or goals that are linked to their own personal values and interests (Donald et al., 2020). Also, Smyth and Milyavskaya (2021) suggest that effects of mindfulness on motivation depend on the personal degree of viewing the corresponding goal as meaningful. Hafenbrack and Vohs (2018) assessed participants’ motivation for an instructed task, but not a task of personal value and interest. Hence, their results may not generalize to tasks and goals of more personal importance.

Based on the above considerations, the present study set out to explore the causal effects of a brief mindfulness intervention on the different forms of motivation according to SDT. We hypothesized that the mindfulness intervention increases motivation towards its more autonomous forms, but that effects are larger for individuals with lower trait mindfulness (Research Hypothesis [RH] 1). Moreover, we predicted that the effects of the mindfulness intervention differ between the various forms of motivation according to SDT, controlling for baseline motivation and trait mindfulness (RH 2).

## Method

### Participants

Data of 91 German-speaking participants were used for this study (58 women; age: *M* = 31.0, *SD* = 13.3, range: 20–70 year). Participants were either of Austrian (*n* = 28), German (*n* = 31) or Italian (*n* = 32; from Southern Tyrol and, hence, German-speaking as well) nationality. About two-thirds of the participants had a degree from a secondary or tertiary education at the time of data collection (see Table 1).

**Table 1:** Sociodemographic sample characteristics of the intervention and control groups

	Intervention (*n* = 43)		Control (*n* = 48)		
Characteristic	*n*	%	*n*	%	*χ*^2^(*df*)
Female sex	29	67.4	29	60.4	0.48(1)
Meditation experience					0.89(2)
Yes	11	25.6	11	22.9	
Yes, but quit meditating	5	11.6	9	18.8	
Never	27	62.8	28	58.3	
Nationality					2.02(2)
Austria	14	32.6	14	29.2	
Germany	17	39.5	14	29.2	
Other (all Italy)	12	27.9	20	41.7	
Highest education					4.49(3)
Apprenticeship	2	4.7	1	2.1	
Secondary education	26	60.4	26	54.2	
Bachelor/Master	11	25.6	20	41.7	
PhD	4	9.3	1	2.1	
Currently studying	25	58.1	21	43.8	1.86(1)
Currently employed	32	74.4	32	66.7	0.65(1)

**Fig. 2:**
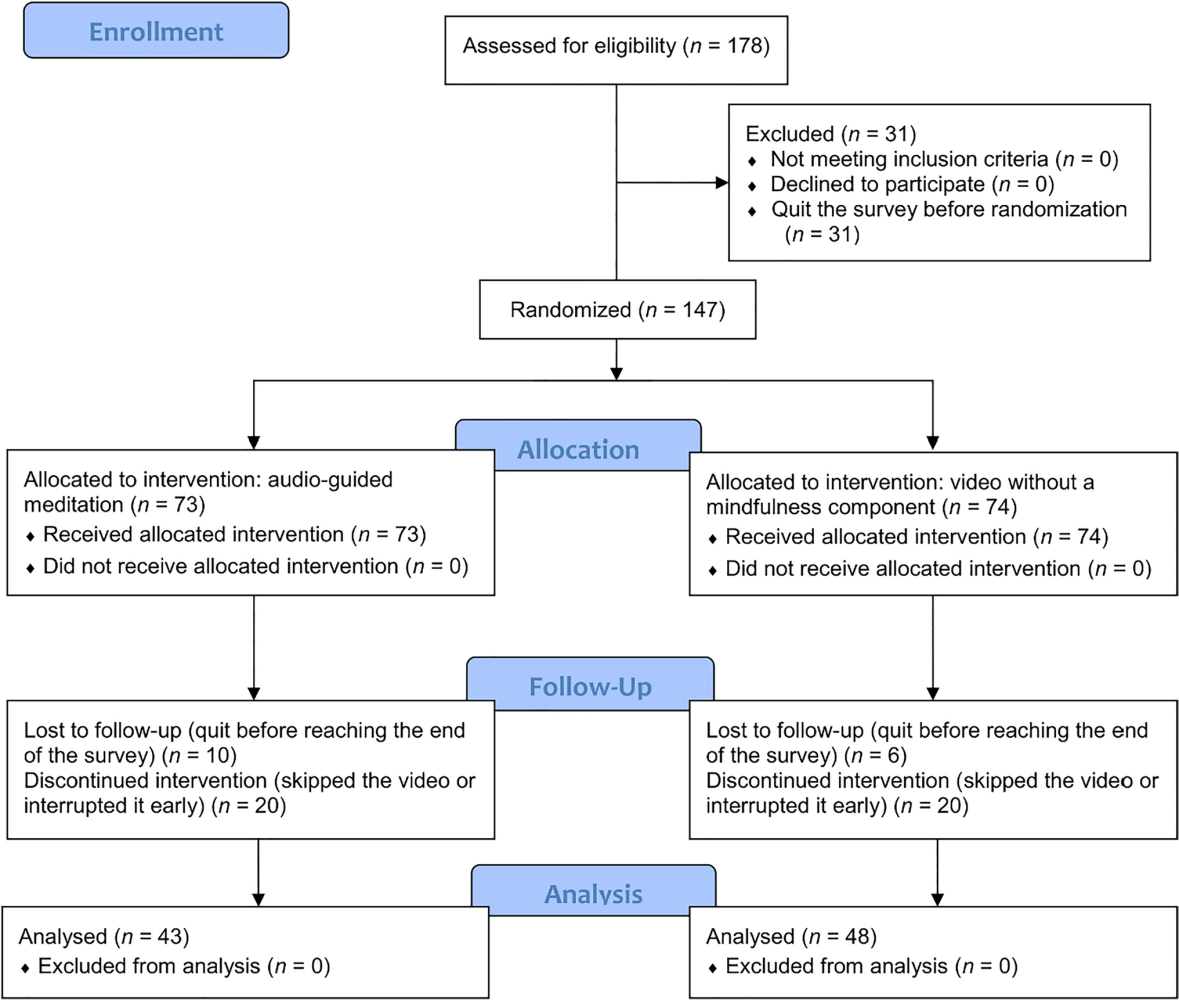
CONSORT flow diagram

Participants were recruited via social media (Facebook, Instagram, WhatsApp) through personal contacts and word of mouth. Participation was voluntary and anonymous, requiring only a minimum age of 18 years and German language skills. Participants provided full informed consent. Due to the COVID-19 pandemic and corresponding lockdown measures, the study was conducted in an online format via SoSci Survey (Leiner, 2019) between April 6 and 14, 2021 (for screenshots of the survey, see https://osf.io/mpbfr/). In total, the link was accessed 417 times. Figure 2 presents the CONSORT flow diagram (see Table S1 in Supplementary Material for the CONSORT checklist). The survey was started by *N* = 178 persons.

### Procedures

Participants were queried for sociodemographic data and meditation experience. Then, trait mindfulness was assessed with the FFMQ-23 (all measures are detailed in the “Measures” section below) and participants were asked about a personal goal they were currently working on and which they would like to achieve within the next 7 days. Following this, the SIMS was used to measure situational motivation in relation to this stated goal. Participants were then randomly assigned to either the intervention or control group. Concealed randomization was performed by an automated algorithm integrated in SoSci Survey. The computerized algorithm utilized a random number generator and conducted equally distributed draws for the two groups (https://www.soscisurvey.de/help/doku.php/en:create:randomization-einfaktoriell). Before reaching randomization, *n* = 31 participants quit the survey.

The remaining *n* = 147 participants were evenly allocated to the intervention and control groups (Fig. 2). The intervention group underwent audio-guided meditation, whereas the control group watched a video without reference to both mindfulness and motivation (details on the intervention and control conditions are provided below). Afterwards, induced state mindfulness was assessed. Finally, situational motivation in regard to the previously stated goal was assessed a second time, using the SIMS.

Of the *n* = 73 participants in the intervention group, a total of *n* = 30 participants were lost to follow-up or had discontinued the intervention (spending between 2 s and 9 min 45 s on the corresponding page of the survey and therefore falling below the predefined minimum watching time of 9 min 50 s; see the following section). In the control group, this number was *n* = 26. The data of all remaining participants were used for analysis (Fig. 2).

In the analysis sample, the average time for completing the questionnaire was 20 min 5 s in the experimental group, whereas 20 min 10 s in the control group. Sample characteristics for the control and intervention groups are presented in Table 1 and show balanced distributions across both groups: the experimental group included *n* = 43 participants (29 women; age: *M* = 31.6, *SD*: 14.5, range: 21–70 year), of whom 16 had any prior meditation experience. The control group included *n* = 48 participants (29 women; age: *M* = 30.5, *SD*: 12.3, range: 20–62 year), of whom 20 had any prior meditation experience. The two groups did not differ in mean age (*t*(89) =  − 0.39, *p* = 0.70, *d* =  − 0.08). Sociodemographic characteristics (as listed in Table 1) did not predict group membership in a multivariate logistic regression analysis either (*χ*^2^ = 10.0, *df* = 9, *p* = 0.35). For this analysis, categories apprenticeship and secondary education, and categories bachelor/master and PhD each were combined in the variable education, as otherwise there were too few data in some categories; participant age was included in this analysis as well.

Sociodemographic characteristics of all *n* = 87 participants who had quit the survey before randomization, who were lost to follow-up, or who had discontinued the intervention were compared with the analysis sample in Table S2 (Supplementary Material). There were no relevant differences between these two groups, except that there were slightly more German nationals in the analysis sample than in the group of excluded participants.

Sociodemographic characteristics did not predict attrition in a multivariate logistic regression analysis though (equal approach as above, *χ*^2^ = 14.2, *df* = 9, *p* = 0.11). Furthermore, participants discontinuing the intervention in the mindfulness or control condition were compared in Table S3 (Supplementary Material); no relevant differences between these groups were observed. 

With the sample size for analysis (*N* = 91), *α* = 0.05 (two-sided), and a desired power of 80%, the smallest detectable between-group effect size would have been *d* = 0.59 (calculated with G*Power; Faul et al., 2007) for the present study. This was well compatible with the magnitude of effects observed in prior related research. In Donald et al. (2020), the meta-analytic effect estimate of mindfulness interventions on identified and intrinsic motivation was *d* = 0.47, 95% *CI* = [0.28, 0.67]; Hafenbrack and Vohs (2018) reported *d*s ranging from 0.30 to 0.72 in their experiments.

#### Brief Mindfulness Intervention

For the induction of state mindfulness, a German audio-guided meditation (“Basics 1”; duration: 10 min 32 s), freely accessible in the meditation app “Headspace” (Headspace Inc., 2021), was used. It aims to help participants getting a focus on the present moment and relaxing their body. The video was not presented in the original app, but rather online on screen. It only showed an orange screen with an inserted countdown of its total length, besides providing an audio track. For the intervention to be considered validly conducted, a minimum watching time of 9 min 50 s was set, since from that point onwards the audio focuses on being present in the environment rather than on meditation itself.

For comparison, a German video (“What do these emojis mean?”; from ProSieben Germany, broadcast in the program Galileo; duration: 10 min 6 s; https://www.youtube.com/watch?v=ZXgw2n4EEsE), which discusses the history of emojis, was shown in the control group. This video neither contained references to mindfulness nor motivational components (independently assessed by two researchers, authors SO and HW). To match the intervention condition, a minimum watching time of 9 min 50 s was set.

### Measures

#### Meditation Experience

Meditation experience was assessed by a total of six items. Participants reported whether they had any meditation experience (*yes*; *no more, I stopped*; or *I have never meditated*) and, if yes, rated the frequency of mindfulness exercises, autogenic training, or progressive muscle relaxation, or any other relaxation technique on 5-point scales (0 = *never*, 1 = *not regularly*, 2 = *at least twice a month*, 3 = *once a week*, 4 = *twice a week*, 5 = *three times a week*, 6 = *four times a week or more*). Regular meditators (score of 2 on any of the above items) also provided the amount of time (in years) of regular practice and the type of meditation or mindfulness exercise most frequently practiced during the last 6 months (Zen, Vipassana, Tai Chi, Qi Gong, Yoga, transcendental meditation, MBSR, other). Information on meditation experience is reported in the present study and was used for baseline comparisons of the intervention and control groups.

#### Trait Mindfulness

A shortened German 23-item version (FFMQ-23; Burzler et al., 2019) of the Five Facet Mindfulness Questionnaire (FFMQ; Baer et al., 2006) was used to assess trait mindfulness. Four items each (scored from 1 = *never true* to 5 = *very often true*) assessed the facets Observe, Describe, Nonjudging of Inner Experience, and Acting with Awareness, whereas all seven items of the original FFMQ assessed the facet Nonreactivity to Inner Experience. Burzler et al. (2019) reported good factorial validity and internal consistency (Cronbach’s *α* ranging from 0.70 to 0.82 for the five facets) for this short form that builds on two earlier forms with slightly different item compositions (Tran et al., 2013, 2014). Even though there is evidence of a two-factor higher order structure in the FFMQ (Burzler et al., 2019; Tran et al., 2013, 2014), total scale scores were utilized, as the present work was interested only in controlling for overall trait mindfulness, but not for the individual aspects of mindfulness separately (exploratory separate analyses for the five facets are, however, presented in Supplementary Material; see the “Results” section). Total scores ranged from 23 to 115, with higher scores signifying higher trait mindfulness. Sample scale reliabilities for Observe, Describe, Actaware, Nonjudge, and Nonreact were 0.67, 0.76, 0.87, 0.76, and 0.90 (Cronbach’s *α*), and 0.66, 0.75, 0.85, 0.76, and 0.89 (McDonald’s *ω*; McDonald, 1999), respectively, using JASP (Love et al., 2019) version 0.14.1 for calculations.

#### Motivation

The German version (Vogt, 2004) of the Situational Motivation Scale (SIMS; Guay et al., 2000) was used to measure situational motivation, assessing Intrinsic Motivation, Identified Motivation, External Motivation, and Amotivation prior to and after the intervention with four items each (scored from 1 = *does not correspond at all* to 7 = *corresponds exactly*). The SIMS has demonstrated good internal consistency in previous research (Cronbach’s *α* ranging from 0.77 to 0.95 for the different subscales; Guay et al., 2000) and factorial validity (Østerlie et al., 2019).

Mean scores for each subscale were calculated, as well as an overall self-determination index (SDI) of situational motivation for which each subscale was weighted according to their position on the self-determination continuum: SDI = 2 * Intrinsic Motivation + Identified Motivation − External Regulation − 2*Amotivation (Paixão et al., 2017). Subscale scores ranged from 1 to 7, with higher scores indicating higher levels of the respective forms of motivation or amotivation, respectively. SDI scores ranged from − 18 to + 18, with higher scores indicating higher situational self-determination (i.e., more autonomous vs. controlled forms of motivation or amotivation). SDI scores were used for testing RH 1, whereas subscale scores for testing RH 2. Sample scale reliabilities for the pre-interventional scores of intrinsic, identified, and external motivation, and amotivation were 0.90, 0.81, 0.79, and 0.73 (Cronbach’s *α*), and 0.90, 0.79, 0.75, and 0.68 (McDonald’s *ω*); reliabilities for the post-interventional scores were 0.91, 0.84, 0.90, and 0.87 (Cronbach’s *α*), and 0.91, 0.82, 0.88, and 0.84 (McDonald’s *ω*), respectively.

#### Induced State Mindfulness

A manipulation check was adopted from Hafenbrack and Vohs (2018) to examine whether the meditation intervention was successful in inducing state mindfulness, relative to the control condition. This manipulation check comprised six items (scored from 1 = *very slightly* to 7 = *extremely*) and was translated from English to German by utilizing the parallel-blind technique (Behling & Law, 2000). Due to the similarity of Items 2 and 3, Item 3 was excluded, so that a total of five items resulted for the manipulation check. A total score was calculated by averaging the item scores. The induction of state mindfulness was considered successful, if there was a significant difference between the intervention and the control groups after the intervention. This comparison served as a manipulation check of our intervention. Sample scale reliability was 0.85 (Cronbach’s *α*) and 0.86 (McDonald’s *ω*), respectively.

### Data Analyses

Analysis proceeded in three main steps. First, independent-groups *t* tests were used to compare situational motivation (SDI) scores and trait mindfulness scores between intervention and control groups at baseline (to assure about their commensurability) and induced state mindfulness scores after the intervention. This latter test served as a manipulation check of the intervention.

Second, to examine RH 1, a hierarchical regression analysis was performed, with post-interventional situational motivation (SDI) scores as the dependent variable. Baseline motivation score (SDI) was entered as a predictor in Model 1, whereas group membership (coded 0 = control group, 1 = intervention group) in Model 2. In Model 3, trait mindfulness was added together with its interaction term with group membership. This interaction term allowed for the investigation of the moderating effect of trait mindfulness on the effect of the mindfulness intervention on motivation. All continuous predictors were centered prior to analysis. Standardized mean differences (conditional effects in the metric of Cohen *d*) at the mean and 1 standard deviation above and below the mean of the moderator (i.e., trait mindfulness) were calculated using formulae presented in Bodner (2017). Note that the conditional effect at the mean is identical to the overall effect of the intervention, when moderation is not considered. As confidence intervals for the conditional effects are unavailable with this approach, we applied the method of Johnson and Neyman (1936) to evaluate at which values of the moderator the intervention effect turned significant.

Third, to examine RH 2, hierarchical regression models similar to the above ones were performed, but using post-interventional SIMS subscale scores as the dependent variables. Again, three models each were fitted to individual post-interventional subscale scores, using the individual baseline subscale scores as predictors in Model 1, group membership in Model 2, and trait mindfulness and its interaction term with group in Model 3. To directly assess whether effects differed between the four types of motivation, we also present in the Supplementary Material the results of a multilevel regression model which simultaneously investigated all four types of motivation and tested for differences between them, utilizing interaction terms.

Analysis was done using IBM SPSS version 27. Python libraries matplotlib (Hunter, 2007), pandas (Reback et al., 2022), and numpy (Harris et al., 2020) were used for graphical displays. Multicollinearity was screened by assessing variance inflation factors (VIFs) of all predictors, using SPSS. For the Johnson-Neyman method, the SPSS macro PROCESS version 4 (Hayes, 2022) was used. For all statistical tests, significance was set to *p* < 0.05 (two-sided).

## Results

### Baseline Comparisons and Manipulation Check

There were no significant baseline differences between the intervention and control groups in baseline situational motivation (SDI) and trait mindfulness (FFMQ-23) scores (Table 2). However, there was a large and significant difference in induced state mindfulness scores. Thus, groups were comparable in situational motivation (SDI) and trait mindfulness at baseline, while the successful manipulation check indicated that the mindfulness intervention indeed raised state mindfulness levels.

**Table 2:** Comparison of baseline motivation (SDI), trait mindfulness, and induced state mindfulness in the intervention and control groups

Measure	Intervention (*n* = 43)	Control (*n* = 48)	*t*(89)	*p*	Cohen *d*	95% *CI*
Baseline situational motivation (SDI)	5.78 (4.79)	6.79 (5.85)	0.90	.37	− 0.19	[− 0.60, 0.26]
Trait mindfulness	77.30 (10.37)	80.38 (12.71)	1.26	.21	− 0.26	[− 0.68, 0.15]
Induced state mindfulness	22.86 (4.99)	18.52 (5.90)	3.77	< .001	0.79	[0.36, 1.22]

*SDI*, self-determination index; *CI*, confidence interval. Numbers are means and standard deviations (in parentheses), unless stated otherwise

### Effects of the Mindfulness Intervention on Situational Motivation (SDI)

**Fig. 3:**
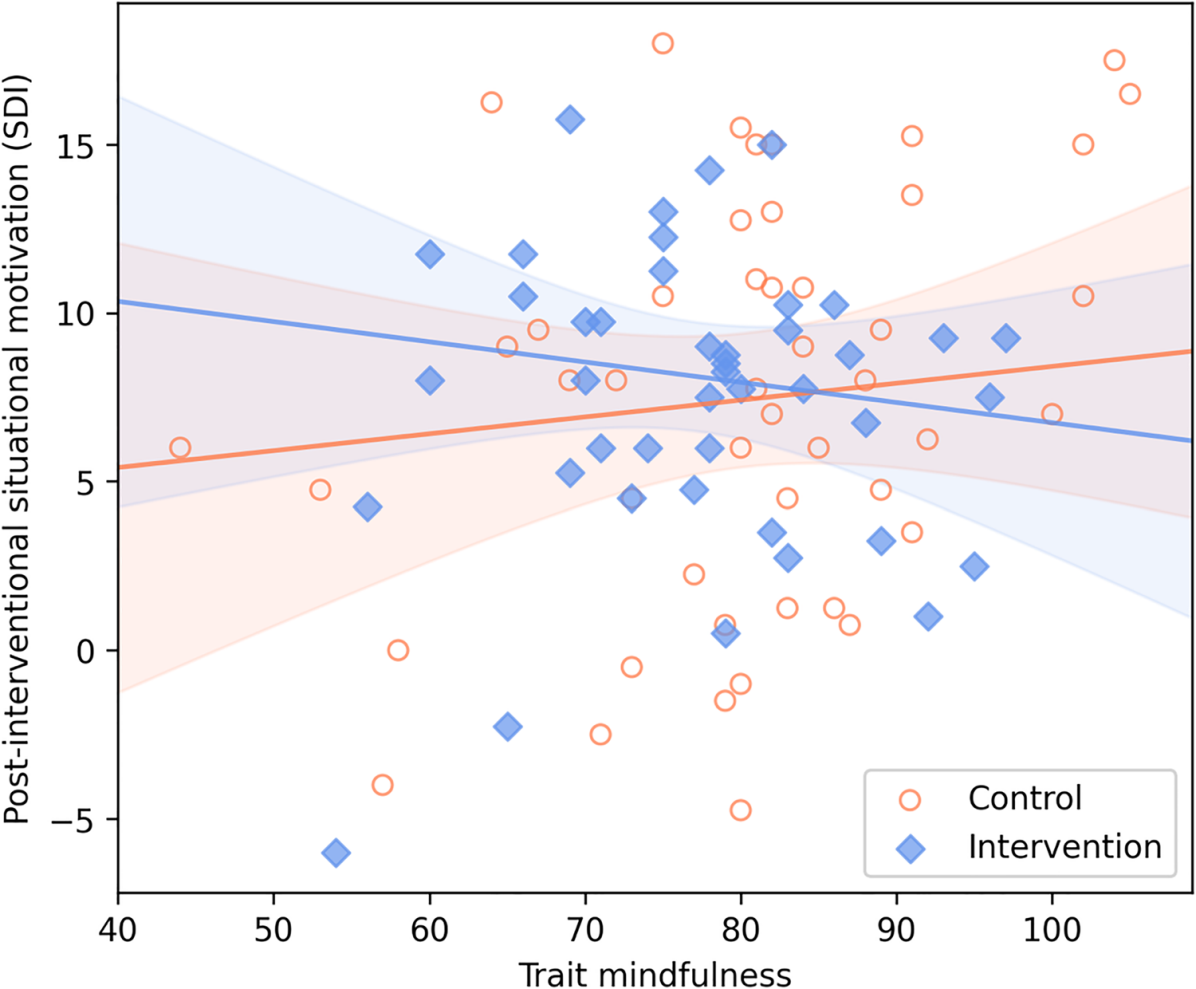
Scatterplot of the intervention group (blue squares) versus control group (orange circles), showing the interaction of group (causal variable) with trait mindfulness (moderator) on post-interventional situational motivation (SDI; outcome). *Note.* The vertical distance between the regression lines of best fit (depicted with corresponding 95% confidence bands) in the intervention and control groups quantifies the intervention effect at each point of the moderator

Table 3 shows the results of the hierarchical regression analysis, and Table S4 presents the pre- and post-interventional means of all motivation scores in the intervention and control groups. Model 1 explained 85% of the total variance (adjusted *R*^2^), whereas Models 2 and 3 86% and 87%, respectively. The differences between Models 1 and 2, and Models 2 and 3 were significant (Δ*R*^2^ = 1%, Δ*F*(1, 88) = 5.34, *p* = 0.02, and Δ*R*^2^ = 1%, Δ*F*(2, 86) = 4.38, *p* = 0.02, respectively). Baseline situational motivation (SDI) positively predicted post-interventional motivation in all three models. The effect of the mindfulness intervention on post-interventional situational motivation (SDI) was medium-sized in Model 2 (*d* = 0.49, 95% *CI* = [0.07, 0.91]), with participants in the intervention group scoring higher than participants in the control group. Yet, Model 3 showed that the effect of the mindfulness intervention was moderated by trait mindfulness (see Fig. 3). Whereas there was a slight positive association of trait mindfulness with post-interventional situational motivation (SDI) in the control group (significant main effect of trait mindfulness in Table 3; see also Fig. 3), the association of trait mindfulness with post-interventional situational motivation (SDI) was negative in the intervention group (see Table 3 and Fig. 3).

**Table 3:** Predicting post-interventional situational motivation (SDI)

Predictor	*B* (*SE*)	*F*(*df*_1_, *df*_2_)	Adjusted *R*^2^
Model 1		522.32(1, 89)***	85%
Baseline situational motivation (SDI)	0.92 (0.04)***		
Model 2		276.58(2, 88)***	86%
Baseline situational motivation (SDI)	0.92 (0.04)***		
Group (intervention vs. control)	0.97 (0.42)*		
Model 3		151.12(4, 86)***	87%
Baseline situational motivation (SDI)	0.92 (0.04)***		
Group (intervention vs. control)	0.93 (0.41)**		
Trait mindfulness	0.05 (0.02)*		
Trait mindfulness × group	− 0.11 (0.04)**		

*SDI*, self-determination index; *B*, unstandardized regression coefficient; *SE*, standard error. For the individual predictors, *t* values can be computed by dividing the provided parameter estimates by their standard errors

^*^p < .05, **p < .01, ***p < .001

This implied that the intervention was successful specifically among participants low in trait mindfulness. The size of the intervention effect was *d* = 1.13 at 1 *SD* below the mean of the moderator, *d* = 0.48 at its mean (which also constituted the intervention effect, not considering moderation, in Model 3), and *d* =  − 0.16 at 1 *SD* above the mean. The Johnson-Neyman method indicated that the intervention effect turned significant (*p* < 0.05) at a value of 79 and below in the moderator, which corresponded to 0.01 *SD* above the mean in the current data. All VIFs of predictors not involving the interaction were smaller than two, thus indicating no relevant multicollinearity in the regression models (Schneider, 2007). Results of Model 3 for each of the five FFMQ facets, controlling for all other facets, are presented in the Supplementary Material (Table S5). These exploratory analyses suggested that the interaction effect can be individually traced especially to the Describe and Nonjudging of Inner Experience facets.

### Effects of the Mindfulness Intervention on the Various Forms of Situational Motivation

Table 4 shows the results of all hierarchical regression analyses (see Table S4 for the individual means of the pre- and post-interventional motivation scores). In Model 2, the intervention effect was statistically significant for identified motivation only (and of medium size), comparable to the magnitude of the intervention effect for situational motivation (SDI) in the foregoing analysis. The intervention effect was descriptively smaller for intrinsic motivation and, directionally opposite, for amotivation; it appeared negligible for external motivation.

**Table 4:** Predicting the different forms of post-interventional situational motivation

Predictor	Intrinsic motivation	Identified motivation	External motivation	Amotivation
Model 1				
Baseline situational motivation^a^	0.92 (0.04)***	0.73 (0.07)***	0.94 (0.05)***	0.77 (0.07)***
* F*(*df*_1_, *df*_2_)	660.579 (1, 89)***	118.23 (1, 89)***	323.13 (1, 89) ***	110.00 (1, 89) ***
Adjusted *R*^2^	88%	57%	78%	55%
Model 2				
Baseline situational motivation^a^	0.92 (0.04)***	0.72 (0.07)***	0.98 (0.06)***	0.77 (0.07)***
Group (intervention vs. control)	0.15 (0.11)	0.31 (0.14)*	− 0.07 (0.19)	− 0.15 (0.11)
* F*(*df*_1_, *df*_2_)	333.78 (2, 88)***	64.41 (2, 88)***	160.11 (2, 88)***	56.48 (2, 88)***
Adjusted *R*^2^	88%	59%	78%	55%
Δ*F*(*df*_1_, *df*_2_)	1.71 (1, 88)	5.09 (1,88)*	0.16 (1,88)	1.87 (1,88)
Δ*R*^2^	< 1%	2%	< 1%	1%
Model 3				
Baseline situational motivation^a^	0.92 (0.04)***	0.71 (0.07)***	0.98 (0.06)***	0.76 (0.07)***
Group (intervention vs. control)	0.14 (0.11)	0.31 (0.14)*	− 0.06 (0.19)	− 0.15 (0.11)
Trait mindfulness	0.01 (0.01)	0.01 (0.01)	− 0.004 (0.01)	− 0.01 (0.01)
Trait mindfulness × group	− 0.02 (0.01)*	− 0.02 (0.01), *p* = .067	0.02 (0.02)	0.02 (0.01)
* F*(*df*_1_, *df*_2_)	176.07 (4, 86)***	33.78 (4, 86)***	79.19 (4, 86)***	28.94 (4, 86)***
Adjusted *R*^2^	89%	59%	78%	55%
Δ*F*(*df*_1_, *df*_2_)	3.02 (2, 86), *p* = .054	1.87 (2, 86)	0.41 (2, 86)	1.18 (2, 86)
Δ*R*^2^	1%	2%	< 1%	1%
Intervention effect (Model 2)	0.28 [− 0.14, 0.69]	0.47 [0.06, 0.89]	− 0.08 [− 0.50, 0.34]	− 0.29 [− 0.71, 0.13]
Conditional effects (Model 3)				
At 1 *SD* below mean	0.80	0.89	− 0.27	− 0.62
At mean of moderator	0.27	0.48	− 0.07	− 0.28
At 1 *SD* above mean	− 0.27	0.07	0.12	0.06

Entries are unstandardized regression coefficients (standard errors in parentheses), unless noted otherwise. Intervention and conditional effects are in the metric of Cohen *d*, with 95% confidence intervals in brackets for the former. For the individual predictors, *t* values can be computed by dividing the provided parameter estimates by their standard errors

^a^Using baseline scores of the same form of motivation as for the post-intervention scores, which were utilized as outcome in each individual analysis

^*^p < .05, **p < .01, ***p < .001

**Fig. 4:**
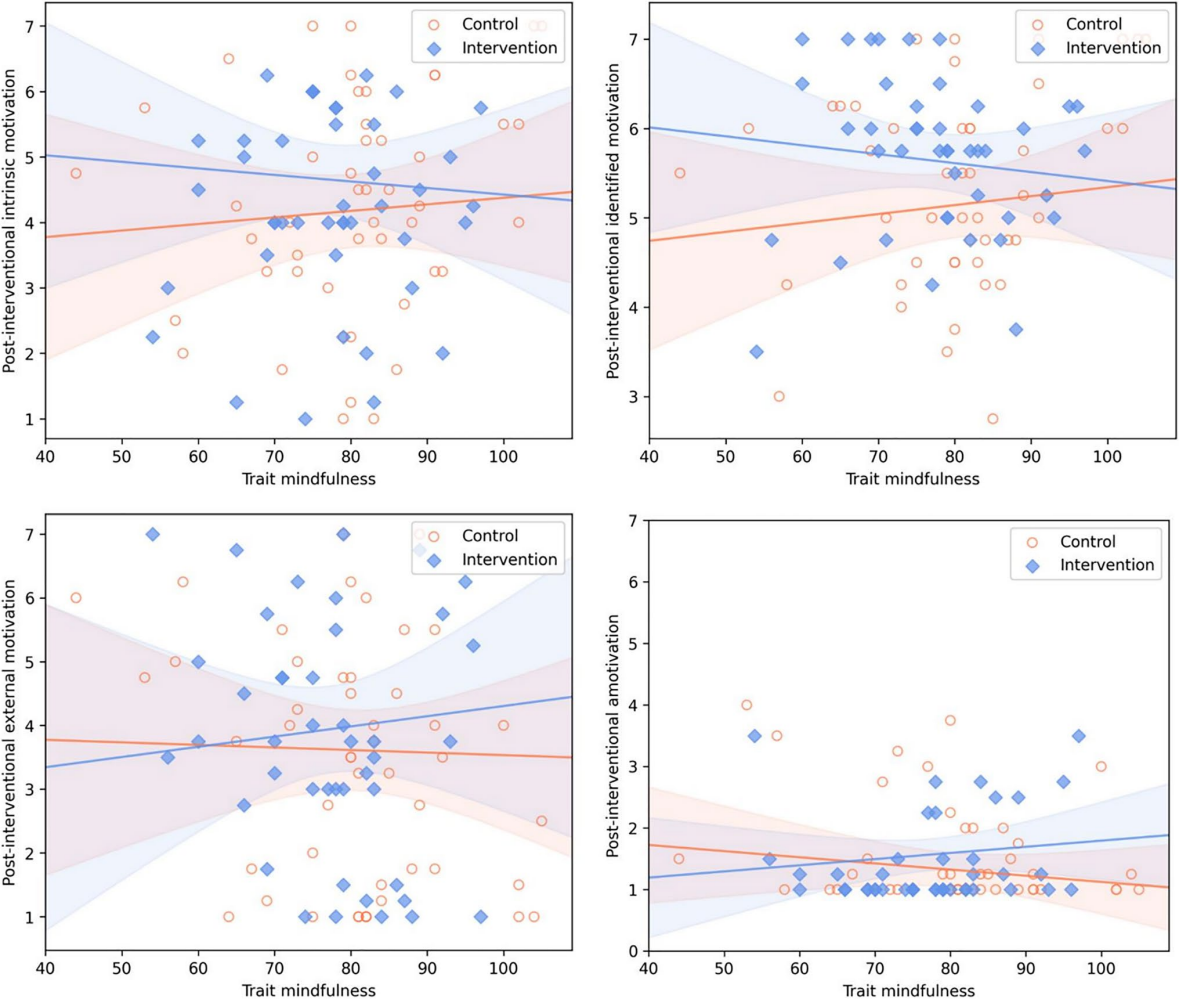
Scatterplots of the intervention group (blue squares) versus control group (orange circles), showing the interactions of group (causal variable) with trait mindfulness (moderator) on the different forms of post-interventional situational motivation (outcomes). *Note.* The vertical distance between the regression lines of best fit (depicted with corresponding 95% confidence bands) in the intervention and control groups in each scatter plot (first to second row and from left to right: intrinsic motivation, identified motivation, external motivation, amotivation) quantifies the intervention effect at each point of the moderator

In Model 3, the interaction of trait mindfulness with the intervention effect was nominally significant only for intrinsic motivation. Yet, albeit statistically not significant, the pattern and magnitude of conditional effects were also similar for identified motivation and, directionally opposite, but of smaller magnitude, for amotivation. Graphical displays of the results are provided in Fig. 4.

Conditional effects of the intervention appeared to be large at 1 *SD* below the mean of the moderator for intrinsic and identified motivation, and medium for amotivation. Conditional effects were smallest, and mostly negligible, for external motivation. The intervention effect turned significant (*p* < 0.05; Johnson-Neyman method) at values of 75 and 80 (− 0.33 *SD* below the mean and 0.09 *SD* above the mean) and below in the moderator for intrinsic motivation and identified motivation, respectively. There were no significant regions of the moderator for external motivation and amotivation. All VIF predictors not involving the interactions were smaller than two, thus not suggesting relevant multicollinearity in the models.

The multilevel analysis (see Table S6 for further details and full information on the statistical model) suggested a significant overall intervention effect that did not differ between types of motivation and a trait mindfulness * group interaction that was statistically significant across all types of motivation combined (*p* = 0.002).

## Discussion

This RCT investigated the effects of a brief mindfulness intervention on motivation, considering different forms of motivation (according to SDT) and individuals’ trait mindfulness and baseline motivation towards a personal goal. Results were consistent with a positive causal effect of mindfulness interventions towards more autonomous forms of motivation, but also indicated that trait mindfulness moderated the magnitude of this effect (RH 1). The results further suggested that the mindfulness intervention mainly affected intrinsic and identified motivation, whereas less so external motivation and amotivation (RH 2). Yet, the results of the multilevel analysis were still compatible with the assumption that all four types of motivation were similarly affected by the intervention (but see our discussion on the limitations of this analysis below). Thus, previously reported negative associations of mindfulness with controlled forms of motivation and amotivation in correlational studies (Donald et al., 2020) could still be causal. Importantly, previous studies did not account for trait mindfulness and mostly also did not account for baseline motivation—variables which turned out important predictors in the present study and, in the case of trait mindfulness, a moderator of the intervention effect.

The findings concerning intrinsic and identified motivation are partly consistent with Donald et al. (2020). They specifically imply that mindfulness interventions may have a positive impact on *identified* goal motives whose values and drives already have been internalized to some extent, but not yet fully. *Intrinsically* motivated goals that already fully correspond to one’s values, beliefs, and principles were also positively affected by the mindfulness intervention, but statistically significantly so only among participants at low levels of trait mindfulness in the individual analyses of motivation type. Furthermore, the present results also provide a new explanation for Hafenbrack and Vohs’ (2018) negative findings. The previous study did not differentiate between personally chosen goals and instructed tasks, which in turn may induce different types of motivation. In the present study, the mindfulness intervention apparently (in the individual analyses) did not increase controlled motivation and decreased amotivation for *personally chosen* goals, while Hafenbrack and Vohs (2018) found a decrease of motivation (undifferentiated for its various forms) for *instructed* tasks. This difference in motivation may have to do with the type of goals (and thus with the corresponding types of motivation). The results of Hafenbrack and Vohs (2018) may correctly describe the situation where goals and tasks have not already been internalized, or probably even cannot be internalized, to a sufficient extent. Yet, the explanation of why these results were obtained in the first place may require the nuance provided here.

We reiterate here that no increases of motivation under such circumstances should actually be deemed a beneficial effect of mindfulness, rather than a downside, as this may benefit mental health and personal growth (e.g., Walach et al., 2007). For example, while autonomous motivation in the workplace was shown to be positively related with job contentment, controlled motivation correlated positively with turnover, but negatively with job satisfaction (Gillet et al., 2013). Yet, more research is still needed here, using a framework of motivation which also differentiates its various forms.

Brown and Ryan (2003) reported positive associations of state and trait mindfulness with autonomous forms of day-to-day activities and well-being. There appears to be broad consensus in the extant literature to differentiate state and trait mindfulness, and moderating effects of trait mindfulness on intervention effects appear logical, given the ramifications of state and trait mindfulness (see Medvedev et al., 2017). However, previous research did not account for them (e.g., Hafenbrack & Vohs, 2018; Smyth & Milyavskaya, 2021).

The present study highlights that high trait mindfulness may limit the effects of mindfulness interventions. This needs to be considered both in the application of mindfulness interventions (greatest benefits are to be expected specifically among the less mindful), but also for their evaluation, like, for example, in the meta-analytic aggregation of efficacy studies and RCTs. Thus, pre-interventional trait mindfulness levels or their correlates, such as age, educational level, or prior meditation experience (Baer et al., 2008), may need to be controlled for in a systematic fashion in the evaluation of mindfulness interventions. Otherwise, the efficacy of these interventions could easily be underestimated. This was demonstrated in the present study, which, like so many other studies in the field of mindfulness research, investigated a mostly highly educated sample and included individuals already (well) acquainted with mindfulness or even experienced in mindfulness meditation.

At the same time, the current results suggest that it could be beneficial to assess baseline mindfulness levels of potential participants before implementing a mindfulness intervention. The intervention could be then offered especially to those who (based on their pre-interventional mindfulness levels) are expected to benefit most. This idea needs to be followed up in future research.

Controlling for the pre-interventional (i.e., baseline) levels of the outcome of interest is common practice in the evaluation of RCTs, as this increases analytic power for testing the effect of interest (i.e., post-interventional differences between intervention and control groups; Van Breukelen, 2006), as well as internal study validity. Doing so also needs to be considered more in studies on the effects of mindfulness interventions on motivation, as highlighted by Smyth and Milyavskaya (2021), who emphasize the necessity of using baseline scores to separate the effects of mindfulness interventions from those of a comparison condition in this field of research.

The COVID-19 pandemic made questions about motivation even more pressing and relevant than before as lockdowns, working in home office, and the loss of obligations and daily structure all pose a threat and challenge to motivation and its different forms. Therefore, the present results appear to have high practical relevance for various aspects of life in the personal, work-related, and the public field. They confirm that even brief mindfulness interventions may boost the more autonomous forms of motivation for one’s personal goals. Brief mindfulness interventions could thus serve as easily applicable and valuable means to help pursuing and achieving one’s goals. Yet, in work environments, wherein employees are mostly motivated through external factors, such as money and benefits, mindfulness interventions might not increase motivation. Future research should explore the effects of mindfulness motivations in such settings, diligently considering the extent of internalization of work-related goals.

This RCT provides further evidence that mindfulness interventions may have a positive impact on different forms of motivation, boosting specifically more autonomous forms. However, there appears to be no causal effect on controlled forms of motivation and amotivation. Consequently, this emphasizes the importance of differentiating between these different forms of motivation. While our results mostly supported recent findings by Donald et al. (2020), they also accentuate the necessity of controlling for baseline motivation and trait mindfulness as moderators of the effects of mindfulness interventions. Mindfulness interventions should be specifically offered to those who might benefit most, i.e., persons low in trait mindfulness. The moderating effects of trait mindfulness may also need to be considered in other lines of inquiry.

### Limitations and Future Research

The present study accounted only for overall trait mindfulness and did not differentiate two higher order factors in the FFMQ (Burzler et al., 2019; Tran et al., 2013, 2014). Previous research has indicated that meditators and non-meditators differ in their structure of trait mindfulness and that a single higher order factor is fully valid for the FFMQ only among meditators. However, the current study included both meditators and non-meditators and was interested mainly in overall levels of mindfulness (but not in the more specific contributions of the two higher order factors or individual facets), and sample size was relatively small (which also limited the number of variables that could be practically modelled in analysis). Future research with larger samples should also differentiate the two higher order factors of mindfulness or analyze its facets in more detail. The use of larger samples could still increase analytic power and precision of effect estimates. Similarly, the multilevel analysis had only relatively low power to detect differences between the four types of motivation. Hence, the results of this analysis have to be treated with caution. Additionally, groups with lower educational level and less prior mediation experience need to be specifically investigated in future studies. This could provide more accurate and better generalizable efficacy estimates of mindfulness interventions.

Response biases (e.g., expectational effects, response-shift effects) cannot be ruled out in the present study and could therefore constitute a risk to its internal validity. Also, results could be subject to common method bias (Podsakoff et al., 2012) as they were solely based on self-report data. Furthermore, participants were asked about their personal goals, which may have influenced the motivational processes involved in achieving them and, hence, also the different forms of motivation which were modeled as outcomes in the present study. Furthermore, as goals were self-selected, the specific goals chosen by participants might have operated as moderators of the effect of the mindfulness intervention. Future research should try to categorize goals and research their possible impact.

The utilized mindfulness intervention was only conducted in one short session. Even though our results imply, and confirm (see Medvedev et al., 2017), that even brief mindfulness interventions may have measurable and relevant effects, our study does not allow for conclusions regarding the effects of longer and more frequent or intensive mindfulness interventions or the duration of observed effects.

Finally, the present study was conducted during the COVID-19 pandemic. This could have impacted participants’ motivation in general, as well as factors that might have influenced their motivation. Also, because the survey was conducted during a lockdown, the data could only be collected online and not in a more controlled lab setting.

## Supplementary Information

Below is the link to the electronic supplementary material.

Supplementary file1 (PDF 204 KB)

## Data Availability

All data, materials, and code to reproduce the analysis are available at the Open Science Framework https://osf.io/mpbfr/.
