# Supplementary material for: No Effects of a Brief Mindfulness Intervention on Controlled Motivation and Amotivation, but Effect Moderation Through Trait Mindfulness: a Randomized Controlled Trial
Source: Mindfulness (N Y). 2022 Aug 24;13(10):2434–47. doi: 10.1007/s12671-022-01968-7 (PMC9399590; doi:10.1007/s12671-022-01968-7)
Supplement: Supplementary file 1 — Supplementary file1 (PDF 204 KB) [file 12671_2022_1968_MOESM1_ESM.pdf]

Supplementary Material

Table S1

CONSORT Checklist

Section/Topic	Item No	Checklist item	Reported on page No
**Title and abstract**			
	1a	Identification as a randomised trial in the title	1
	1b	Structured summary of trial design, methods, results, and conclusions (for specific guidance see CONSORT for abstracts)	3
**Introduction**			
Background and objectives	2a	Scientific background and explanation of rationale	4-7
	2b	Specific objectives or hypotheses	8
**Methods**			
	3a	Description of trial design (such as parallel, factorial) including allocation ratio	12
	3b	Important changes to methods after trial commencement (such as eligibility criteria), with reasons	/
Participants	4a	Eligibility criteria for participants	11
	4b	Settings and locations where the data were collected	11-12
Interventions	5	The interventions for each group with sufficient details to allow replication, including how and when they were actually administered	11
Outcomes	6a	Completely defined pre-specified primary and secondary outcome measures, including how and when they were assessed	10-12
	6b	Any changes to trial outcomes after the trial commenced, with reasons	/
Sample size	7a	How sample size was determined	13-14
	7b	When applicable, explanation of any interim analyses and stopping guidelines	/
Randomisation:			
Sequence generation	8a	Method used to generate the random allocation sequence	12
	8b	Type of randomisation; details of any restriction (such as blocking and block size)	12

Allocation concealment mechanism	9	Mechanism used to implement the random allocation sequence (such as sequentially numbered containers), describing any steps taken to conceal the sequence until interventions were assigned	12
Implementation	10	Who generated the random allocation sequence, who enrolled participants, and who assigned participants to interventions	12
Blinding	11a	If done, who was blinded after assignment to interventions (for example, participants, care providers, those assessing outcomes) and how/	/
	11b	If relevant, description of the similarity of interventions	11
Statistical methods	12a	Statistical methods used to compare groups for primary and secondary outcomes	14-15
	12b	Methods for additional analyses, such as subgroup analyses and adjusted analyses	/
**Results**			
Participant flow (a diagram is strongly recommended)	13a	For each group, the numbers of participants who were randomly assigned, received intended treatment, and were analysed for the primary outcome	12-13
	13b	For each group, losses and exclusions after randomisation, together with reasons	12-13
Recruitment	14a	Dates defining the periods of recruitment and follow-up	11
	14b	Why the trial ended or was stopped	/
Baseline data	15	A table showing baseline demographic and clinical characteristics for each group	29
Numbers analysed	16	For each group, number of participants (denominator) included in each analysis and whether the analysis was by original assigned groups	12-13
Outcomes and estimation	17a	For each primary and secondary outcome, results for each group, and the estimated effect size and its precision (such as 95% confidence interval)	15-17
	17b	For binary outcomes, presentation of both absolute and relative effect sizes is recommended	/
Ancillary analyses	18	Results of any other analyses performed, including subgroup analyses and adjusted analyses, distinguishing pre-specified from exploratory	/
Harms	19	All important harms or unintended effects in each group (for specific guidance see CONSORT for harms)	/
**Discussion**			
Limitations	20	Trial limitations, addressing sources of potential bias, imprecision, and, if relevant, multiplicity of analyses	21-22
Generalisability	21	Generalisability (external validity, applicability) of the trial findings	19 -21
Interpretation	22	Interpretation consistent with results, balancing benefits and harms, and considering other relevant evidence	17-19
**Other information**			

Registration	23	Registration number and name of trial registry	/
Protocol	24	Where the full trial protocol can be accessed, if available	/
Funding	25	Sources of funding and other support (such as supply of drugs), role of funders	2

Table S2

Sample Characteristics of Included versus Excluded Participants

	Included (n = 91)		Excluded (n = 87)		
Characteristic	*n*	%	*n*	%	$\chi^2(df)$
Sex					
Female	58	65.7	49	56.3	0.33(1)
Missing			15	17.2	
Meditation Experience					1.18(2)
Yes	22	24.2	19	21.8	
Yes, but quit meditating	14	15.4	14	16.1	
Never	55	60.4	36	41.1	
Missing			18	20.7	
Nationality					9.68(2)*
Austria	28	30.8	38	43.7	
Germany	31	34.1	12	13.8	
Other	32	35.2	22	25.3	
Missing			15	17.2	
Highest education					5.73(4)
Compulsory school	0	0.0	2	2.3	
Apprenticeship	3	3.3	0	0.0	
Secondary education	52	57.1	41	47.1	
Bachelor/Master	31	34.1	27	31.0	
PhD	5	5.5	2	2.3	
Missing			15	17.2	
Currently Studying	46	49.5	43	49.4	1.62(1)
Missing			16	18.4	
Employment Status	64	70.3	50	57.5	0.00(1)
Missing			16	18.4	

Note. $^*p < .05$.

Table S3

Sample Characteristics of Participants Discontinuing Intervention, Comparing the Intervention and Control Groups

	Intervention (n = 20)		Control (n = 20)		
Characteristic	n	%	n	%	$\chi^2(df)$
Sex					
Female	15	75	13	65	0.47(1)
Meditation Experience					2.74(1)
Yes	8	40	5	25	
Yes, but quit meditating	2	30	6	10	
Never	10	45	9	50	
Nationality					0.46(2)
Austria	11	55	9	45	
Germany	2	10	3	15	
Other	7	35	8	40	
Highest education					2.55(4)
Compulsory school	1	5	1	5	
Apprenticeship	1	5	2	10	
Secondary education	8	40	10	50	
Bachelor/Master	10	50	6	30	
PhD			1	5	
Currently Studying	14	70	12	60	0.44(1)
Employment Status	13	65	16	80	1.12(1)

Table S4

Motivation Means Before and After the Intervention for the Intervention and Control Groups

	Intervention (*n* = 43)		Control (*n* = 48)	
Motivation	T0	T1	T0	T1
SDI	5.77	7.48	6.79	7.45
Intrinsic	3.94	4.23	4.14	4.26
Identified	5.50	5.70	5.41	5.33
External	4.33	3.77	3.86	3.39
Amotivation	1.64	1.45	1.52	1.51

Note. SDI = self-determination index (Paixão et al., 2017).

Table S5

Facets of the FFMQ predicting Post-Interventional Situational Motivation

Predictor	Observe	Describe	Nonjudging	Acting with Awareness	Nonreacting
Model 3					
Baseline situational motivation	0.91 (0.04)***	0.92 (0.04)***	0.93 (0.04)***	0.92 (0.04)***	0.92 (0.04)***
Group (intervention vs. control)	0.95 (0.44)*	0.93 (0.42)*	1.00 (0.43)*	0.96 (0.44)*	0.98 (0.44)*
Observe	0.15 (0.13)	0.03 (0.09)	0.06 (0.09)	0.01 (0.09)	0.01 (0.09)
Describe	0.00 (0.09)	0.24 (0.11)*	-0.04 (0.09)	-0.02 (0.09)	-0.03 (0.09)
Nonjudging of Inner Experience	-0.003 (0.10)	-0.05 (0.09)	0.10 (0.11)	-0.01 (0.09)	-0.05 (0.09)
Acting with Awareness	-0.002 (0.09)	0.02 (0.08)	0.06 (0.08)	0.14 (0.10)	0.02 (0.09)
Nonreacting to Inner Experience	-0.01 (0.06)	-0.02 (0.06)	-0.01(0.06)	0.004 (0.06)	0.10 (0.08)
Trait mindfulness facet in focus * group	-0.26 (0.20)	-0.52 (0.17)**	-0.30 (0.14)*	-0.25 (0.14)	-0.15 (0.09)
$F(df_1, df_2)$	66.59(8, 82)***	73.76(8, 82)***	69.45(8, 82)***	67.76(8, 82)***	67.34(8, 82)***
Adjusted R^2	85%	86%	86%	85%	86%

Note. Entries are unstandardized regression coefficients (standard errors in parentheses), unless noted otherwise. For the individual predictors, t values can be computed by dividing the provided parameter estimates by their standard errors.
*$p < .05$, **$p < .01$, ***$p < .001$.

Table S6

Predicting all Four Types of Motivation in a Multilevel Model, Testing for Differences Between the Four Types

Predictor	*B* (*SE*)	*p*
**Type of motivation (intercept; identified motivation)**	**5.35 (0.10)**	**<.001**
**Intrinsic**	**-1.18 (0.14)**	**<.001**
**External**	**-0.96 (0.14)**	**<.001**
**Amotivation**	**+1.09 (0.14)**	**<.001**
**Baseline motivation (identified motivation)**	**0.71 (0.07)**	**<.001**
**Intrinsic**	**+0.21 (0.08)**	**.011**
**External**	**+0.27 (0.08)**	**.001**
Amotivation	+0.04 (0.12)	.705
**Group (intervention vs. control; identified motivation)**	**0.31 (0.14)**	**.029**
Intrinsic	-0.17 (0.20)	.399
External	-0.24 (0.20)	.220
Amotivation	-0.16 (0.20)	.415
Trait mindfulness (identified motivation)	0.01 (0.01)	.126
Intrinsic	-0.001 (0.01)	.890
External	-0.01 (0.01)	.483
Amotivation	-0.005 (0.01)	.668
Trait mindfulness * group (identified motivation)	-0.02 (0.01)	.073
Intrinsic	-0.002 (0.02)	.914
External	+0.01 (0.02)	.669
Amotivation	+0.01 (0.02)	.678

Note. The multilevel model included all four types of motivation as outcome variables (T1 scores) and predictors (T0 scores). External motivation and amotivation scores were reversed in polarity to align them in direction with the remaining other two types of motivation. All continuous predictors were centered before analysis. In the model, the four types of motivation were differentiated by using three dummy variables, utilizing identified motivation as the common comparator. The model also included interactions of these dummy variables with the other predictors. All lines reading "identified motivation" present parameter estimates for the common comparator. All other lines refer to the respective other three types of motivation, compared to this common comparator, i.e., they present deviations to the respective parameter estimates of the common comparator. The restricted maximum likelihood method was used for parameter estimation, applying a Satterthwaite approximation to the degrees of freedom for the statistical tests of each parameter or deviation. Significant ($p < .05$) model terms are printed boldface.

The results suggested that (1) the various types of motivation differed significantly in mean levels from the common comparator, identified motivation; (2) baseline motivation had a significant effect on each type of motivation – this effect was significantly even stronger for intrinsic and external motivation than identified motivation; (3) there was a significant overall intervention effect that did not differ between types of motivation; (4) trait mindfulness had no significant main effect on any type of motivation; (5) there was some indication (though not statistically significant, $p = .073$) of a trait mindfulness * group interaction that did not differ between types of motivation. However, collapsing this interaction across all types of motivation to increase the analytical power of the model, the trait mindfulness * group interaction was statistically significant ($B = -0.02$, $SE = 0.006$, $p = .002$).

Manipulation-Check Items

Original contents (Hafenbrack & Vohs, 2018)

Please answer the following questions in terms of how you are feeling RIGHT NOW.
Scale: 1 (*not much at all*) to 7 (*extremely*)

1. To what extent are you focused on the present moment right now?
2. To what extent are you focused on your breathing right now?
3. To what extent are you focused on the physical sensations of your breath right now?
4. To what extent are you in touch with your body right now?
5. To what extent are you absorbed in the present moment right now?
6. To what extent are you thinking about the present moment right now?

German translation (using the parallel-blind technique; Behling & Law, 2000)

Bitte beantworten Sie die folgenden Fragen in Bezug darauf, wie Sie sich JETZT GERADE fühlen.
Skala: 1 (*so gut wie gar nicht*) bis 7 (*extrem*)

1. In welchem Ausmaß konzentrieren Sie sich jetzt gerade auf den gegenwärtigen Moment?
2. In welchem Ausmaß konzentrieren Sie sich jetzt gerade auf Ihre Atmung?
   [In welchem Ausmaß konzentrieren Sie sich jetzt gerade auf die körperlichen Empfindungen Ihres Atems?; former Item 3, excluded for its similarity to Item 2]
3. In welchem Ausmaß sind Sie jetzt gerade in Kontakt mit Ihrem Körper?
4. In welchem Ausmaß sind Sie jetzt gerade in den gegenwärtigen Moment vertieft?
5. In welchem Ausmaß denken Sie jetzt gerade über den gegenwärtigen Moment nach?

References

Behling, O., & Law, K. S. (2000). *Translating questionnaires and other research instruments: Problems and solutions.* Sage. https://dx.doi.org/10.4135/9781412986373

Hafenbrack, A. C., & Vohs, K. D. (2018). Mindfulness meditation impairs task motivation but not performance. *Organizational Behavior and Human Decision Processes*, *147*, 1-15. https://doi.org/10.1016/j.obhdp.2018.05.001

Paixão, O., Gamboa, V., & Valadas, S. (2017). Validation of a Portuguese version of the Situational Motivation Scale (SIMS) in academic contexts. *Avances en Psicología Latinoamericana*, *35*(3), 547-557. https://doi.org/10.12804/revistas.urosario.edu.co/apl/a.4767
